# Ease of intubation: King vision video laryngoscope versus macintosh laryngoscope

**DOI:** 10.6026/973206300211234

**Published:** 2025-05-31

**Authors:** Urmila Keshari, Rajni Thakur, Sherin Soni, Ajay Yadav

**Affiliations:** 1 Department of Anaesthesia, Gandhi Medical College, Madhya Pradesh, India

**Keywords:** Macintosh laryngoscope, king vision video laryngoscope, intubation

## Abstract

The ease of endotracheal intubation between the King Vision Video Laryngoscope (KVVL) and the Macintosh Laryngoscope (MAC-L) in 100 adult patients is of interest. Key outcomes assessed were hemodynamic changes, number of intubation attempts, procedure duration, and complications. Results indicated the KVVL had a higher first-attempt success rate (80% vs. 70% for MAC-L), required fewer attempts, and was associated with more stable hemodynamics. No major complications were observed with either device. The study concludes that the King Vision Video Laryngoscope offers advantages in ease of intubation, success rate, and hemodynamic stability over the Macintosh laryngoscope.

## Background:

The most effective method of securing an airway during tracheal intubation was by using the Macintosh laryngoscope (MAC), direct laryngoscopy is the primary technique for tracheal intubation [1]. Teaching this skill is difficult since Macintosh direct laryngoscope (DL) allows only one individual to view the larynx during the procedure [2]. This skill is difficult to master and has long learning curve [3, 4]. Most of the difficult intubation is unpredicted and predictive tests have low sensitivity and positive predictive value [5, 6]. There is no single factor to predict the existence of a difficult airway, so difficult airway is not known until the induction of anaesthesia [7]. Video laryngoscopes had been proven useful for intubating patients with difficult airway by inexperienced laryngoscopists and advocated by experts also. A recently developed video laryngoscope, King Vision laryngoscope can securely and effectively manage both normal and challenging airways. Therefore, it is of interest to evaluate ease of tracheal intubation and time taken for intubation by junior residents with the use of king vision video laryngoscope and Macintosh laryngoscope.

## Materials and Methods:

The present study is Observational hospital based study conducted in the Department of Anaesthesiology, Gandhi medical college and associated Hamidia Hospital from January 2023 to January 2024. After approval from institutional ethics committee under letter number (32293/MC/IEC/2022), and with CTRI number: CTRI/2023/07/055842 study was conducted on 100 patients of ASA grade 1 and 2, aged between 18-60 years schedule for elective surgery, that require general anesthesia, laryngoscopy and tracheal intubation.

## Inclusion criteria:

[1] Patients of ASA grade - I, ASA grade II

[2] Age group 18-60 years of either sex.

[3] BMI <30

[4] MPG - 1 & 2

## Exclusion criteria:

[1] Patient refusal or not giving consent.

[2] Limited oral opening

[3] Limited cervical extension

[4] Short thyro mental distance

[5] Mpg - 3 & 4

[6] Pregnant women

[7] Patient with Co morbidities

[8] Seizure disorder

[9] ASA grade III,IV,V

## Methods:

Preoperatively patients were explained about the procedure and technique and informed consent was taken, each patient was assessed for difficult airway and previous intubation attempts. Baseline parameters of patients such as heart rate, NIBP, mean arterial pressure, spo2, ECG were noted down and monitored accordingly during procedure. General anaesthesia technique was standardised for all patients Premedication was done with inj. glycopyrrolate I.V (0.01mg/kg), inj. fentanyl I.V (1-2mg/kg), inj. midazolam I.V (0.05-0.1mg/kg) done, induction with inj. propofol I.V (2-3mg/kg), inj. succinyl choline I.V (1-2mg/kg) was done. The choice of laryngoscope blade size was at discretion of attending anaesthesiologists. Patients were divided into two groups: Group MAC (n=50) patients intubated by using a macintosh laryngoscope, positioned in sniffing position and intubated with Macintosh laryngoscope. Group KVVL (n=50) patients were intubated by using a king vision video laryngoscope, positioned with head in neutral position and intubated with King vision Laryngoscope, using channeled blade with the endotracheal tube preloaded and entering through midline and requires depression of the tongue, not deviation as with Macintosh laryngoscope. A maximum of two attempts or 120 seconds were allowed with the assigned laryngoscope, if failed to intubate, the patient was ventilated and intubated with conventional laryngoscope by an trained anesthesiologist. All intubations were performed by junior residents of mean age group of 25 years, having no experience in laryngoscopy/intubation/anaesthesiology. We measured the outcome by intubation difficulty score which constitutes 7 different variables associated with difficult intubation and each variable has 0-1 score (Table 1). Time taken to endotracheal intubation defined as time from passage of laryngoscope tip past incisors to the appearance of ETco2 tracing. After extubation, patient was assessed for any airway trauma or cervical spine injury due to hyperextension of neck.

## Data collection procedure:

After taking consent for study protocol, patient was registered for the study. Data was collected by an independent person and entered in the attached patient preform and finally entered in the master chart attached.

## Statistical analysis:

Parametric data was analyzed using student t-test. Non-parametric data was analysed using Mann Whitney 'U' test. Statistical analysis was performed using Statistical Package for Social Sciences® software (SPSS) version 25.0 IBM.

## Results:

In the present observational study 100 patients were divided into two groups.

[1] Group MAC (n=50) patients intubated by using a macintosh laryngoscope.

[2] Group KVVL (n=50) patients were intubated by using a king vision video laryngoscope.

Patients in both the groups were comparable in terms of Age, Gender, BMI and ASA grade, no statistical significance was observed in Age, Gender, BMI and ASA grade (Table 2). There are statistical significant differences in mean heart rates between Groups MAC and KVVL during the all observed time intervals except at 5 minutes time interval following endotracheal intubation. By comparing the two groups according to the MAP, there was a significant decrease in MAP after intubation using the KVVL compared with the MAC-L at time intervals of 15, 20, 30, 60 min with p-values <0.05 after intubation. By comparing the two groups according to the Time taken for intubation (TTI) by novice intubators, there was no significant difference in TTI using the KVVL compared with the MAC-L. By comparing the two groups according to the NUMBER OF ATTEMPTS taken by novice intubators, there was a significant decrease in AVERAGE number of attempts taken for the endotracheal intubation by using the KVVL compared with the MAC-L. By comparing the two groups according to the MAP, there was a significant decrease in MAP after intubation using the KVVL compared with the MAC-L at time intervals of 15,20,30,60 min as shown in Figure 2 - (see PDF) with p-values <0.05 after intubation. Group KVVL and Group MAC had similar baseline heart rates, but significant differences emerged post-intubation, with Group KVVL showing higher heart rates at most time points except at 5 minutes as shown in Figure 1 - (see PDF).

By comparing the two groups according to the Time taken for intubation (TTI) by novice intubators as shown in Table 3, there was no significant difference in TTI using the KVVL compared with the MAC-L. By comparing the two groups according to the NUMBER OF ATTEMPTS taken by novice intubators as shown in below Table 4, there was a significant decrease in AVERAGE number of attempts taken for the endotracheal intubation by using the KVVL compared with the MAC-L. In both the groups, the reason for 2nd and 3rd attempt was difficulty in placement of endotracheal tube. In MAC-L group students faced difficulty in lifting and cricoid compression was also applied. In King Vision laryngoscope the presence of the guiding channel requires a rotational movement rather than external laryngeal manipulation for glottis insertion. As depicted in Figure 3 - (see PDF), Degree of difficulty was less seen in KVVL group intubators with 44% of patients has IDS score of 0 & 1 Each, comparably in mac group where only 22% and 8% was present with this IDS scores. Total number of patients with IDS score of >5 is 0 that infers there was no patient with severe difficulty. Comparably, the mean IDS SCORE of KVVL group (0.79) was much less than MAC group (1.81). Hence, degree of difficulty faced during laryngoscopy and intubation was easy in KVVL group comparable to MAC group. As shown in the below Table 5, the percentage of patients which were intubated at 1st attempt in MAC group and KVVL group were 70% and 80% respectively and at 2nd attempt in MAC group and KVVL group were 26% and 20% respectively and at 3rd attempt in MAC group and KVVL group were 2% and 0% respectively.

## Complications:

There were no major complications as such in any of the 2 groups. The complications anticipated were trauma to lips, teeth, oral cavity, other airway structures and any desaturation events. Other complications such as hoarseness of voice and bleeding from trauma to airway structures were anticipated and 1 patient in each group had soreness in the throat after extubation which got perished within few hours.

## Discussion:

Macintosh laryngoscopes continue to be the most widely used laryngoscope in anesthesiology, despite the fact that there are now several distinct varieties of video laryngoscopes with differing technological specifications and operational features. Though the indirect image produced by the most recent laryngoscopes makes for a better view of the larynx through optical equipment, it also necessitates more adept and-eye coordination throughout the surgery. We conducted an observational study in elective surgery patients requiring general anesthesia with endotracheal intubation using a King Vision video laryngoscope and a Macintosh direct laryngoscope to assess the ease of intubation among junior residents. In our study, the demographic profile was compared between patients of Group MAC and KVVL, results found were not significant (p value>0.005). We observed that mean BMIs of group MAC and KVVL was 21.35 and 21.37 kgm-2, respectively. The percentages of males were more than the females by 4 %. There are no statistically significant differences in both the groups with respect to [Age, Gender, BMI or ASA physical status classification]. The present study showed that, Just 1 min after intubation, there was increase in the heart rate in both groups. But there was a significant difference in the mean heart rates compared to the baseline heart rate more in the MAC group than in KVVL group p-value (0.001) & at subsequent time intervals the mean heart rates are on higher side in MAC group than in KVVL group, reflecting the physiological responses to intubation. Mean arterial pressure (MAP) and heart rate (HR) increased less with KVVL than with the Macintosh laryngoscope, according to study done by Ali *et al.* [8]. In addition, Elhadi *et al.* [9] discovered that the Macintosh group had significantly higher HR and MAP than the KVVL group. In our study, When comparing the KVVL to the MAC-L during intubation, there was a statistically significant drop in mean arterial pressure (MAP) in MAC group at intervals of 15, 20, 30, and 60 minutes, with p-values <0.05. This divergence remained throughout the surgical phase, indicating that the type of laryngoscope used for endotracheal intubation had a major influence on hemodynamics. Compare to traditional direct laryngoscopy, Ralph *et al.*'s study, [10] which used indirect video laryngoscopy with a Macintosh blade video laryngoscope, showed fewer hemo-dynamic reactions during endotracheal intubation. Comparing video laryngoscopy to traditional direct laryngoscopy, the relative rise in the rate pressure product (RPP) at intubation was much reduced (*i.e.*, 27%, P < 0.001).

In our study, residents' mean intubation times utilizing the KVVL (52 seconds) and the MacIntosh laryngoscope (55 seconds) were nearly identical. These were some important findings that led to a longer TTI. Students took longer time to view the glottis and elevate the epiglottis correctly because they lacked laryngoscope handling experience. Murphy *et al.*'s study, [11] states that in the typical manikin airway scenario, the KVVL took 3.4 s (99% confidence interval [CI] 0.1-6.6) less time to intubate compared to the DL. In the challenging cadaver airway scenario, the time to intubation was 11.3 s (99% CI 2.4-20.2) faster when using the KVVL. But the doctors who intubated the patients in their study were experienced in treating airways beforehand. According to Narang *et al.* [12] every participant was able to imitate a difficult airway and intubate a mannequin with typical neck mobility in the allotted time. In the meantime during our study, KVVL was also utilized as a teaching tool during intubation, and we anticipated that by employing KVVL, students would acquire intubation skills more quickly. A study on beginner learners by Low *et al.* [13] found that training with a video laryngoscope improves intubation competence and promotes confidence in a simulated difficult airway scenario, in contrast to a standard Macintosh laryngoscopy. Elhadi *et al.* [9] reported that the average time for inserting a tube was 19.10±7.08 for the Mac-L group and 17.34 ± 4.62 for the KVVL group. This indicates that there was no discernible variation in the intubation insertion time using the two laryngoscopes. In an airway management course for beginners, teaching video laryngoscopy alone utilizing KVVL may be adequate to improve global skill, per Wolf *et al.*'s study [14]. They also found that utilizing KVVL resulted in fewer esophageal intubations and greater skill transfer from KVVL to Macintosh blade than vice versa. Two groups ease of intubation was measured using the intubation difficulty score (IDS): the MAC Group and the KVVL Group. By comparing the Mean IDS scores of 1.81 and 0.79, respectively, between the KVVL and MAC groups, our study's findings indicate that the kvvl group of intubators had less trouble with intubation than the latter. The Intubation Difficulty Score was used by Loughnan *et al.* [15] --1 (0-2) for direct laryngoscopes and -0 (0-1.5) for video laryngoscopes with a 95% confidence interval (p value of 0.13). The IDS score turned out to be statistically insignificant. According to Ali *et al.* [8] the King Vision video laryngoscope group's intubation difficulty scores were significantly lower than those of the other groups (P=0.001). Compared to just 6 patients in the Macintosh group and 8 patients in the McCoy group, 13 patients in the King Vision video laryngoscope group experienced simple intubation (IDS score = 0). In our study, KVVL had a higher first attempt success rate (80%) than MAC-L (70%). Compared to the mac group, there was no incidence of esophageal intubation in the kvvl group. Three attempts were required for tracheal intubation in the 2% of esophageal intubations in the Mac group. According to Wolf *et al.* [14] the success rate with the Macintosh blade was 48%, but the KVVL had a higher rate of 52% (OR 0.85, 95% CI 0.4-2.0). Additionally, there was a statistically significant difference in the rate of esophageal intubations-4% with KVVL and 17% with MAC. First pass intubation success rates for the Macintosh and King Vision were comparable, according to study by Erdivanli *et al.* [16] (94.3% vs. 96.6%, respectively, p > 0.05). According to Ali *et al.* [8] the King Vision video laryngoscope group had a first attempt success rate of 97%, while the Macintosh group had an 86% rate and the McCoy group had a 90% rate. A study by Akihisa *et al.* [1] found no appreciable difference between the KVVL and MAC's success rates.

## Conclusion:

The King Vision Video Laryngoscope is better in terms of ease of intubation, 1st attempt success rate, total time taken for intubation with lesser complications KVVL might be a useful tool for teaching airway novices for intubations and it is better in glottis visibility/laryngeal view when compared to Macintosh laryngoscopy. Further, more training for the King vision video laryngoscope is required to determine its usefulness in difficult airway situation.

## Limitation:

Limitation of this study was lessser sample sizes and exclusion criteria limitations. Further future studies of randomised controlled trials of least possible limitations, with standardised outcome measures to provide more robust evidence must be done to enable better decision making regarding laryngoscope selection for junior residents.

## Figures and Tables

**Table 1:** Intubation difficulty score

**IDS PARAMETER**		**SCORE**
Number of attempts > 1		N1
Number of attempts > 1		N2
Number of alternative techniques		N3
Cormack and lehane grade - 1		N4
Lifting force required		
Normal		N5 =0
Greater than normal		N5=1
Cricoid compression		
Not applied		N6=0
applied		N6=1
Vocal cord mobility		
Abduction		N7=0
Adduction		N7=1
TOTAL:IDS= SUM OF SCORES		N1-N7
**IDS SCORE**	**Degree of Difficulty**	
0	Easy	
0<IDS<5	Slight difficulty	
IDS >5	Moderate to major difficulty	

**Table 2:** Demographic data

**Parameters**	**Group MAC (n=50)**	**Group KVVL (n=50)**	**P-value**
Age (years)	37.85 ±12.44	35.12± 12.47	0.275
BMI( kgs/m^2^)	21.35 ± 2.24	21.37 ± 2.635	0.96
sex (M/F)	27/23	25/25	0.688
ASA Grade (1/2)	21/29	27/23	0.229

**Table 3:** Comparison of mean time taken for intubation

**Variable**	**MAC GROUP**	**KVVL GROUP**
MEAN TIME TAKEN FOR INTUBATION	55 SECONDS	52 SECONDS

**Table 4:** Comparison of mean number of attempts taken for intubation in between the groups

**Variable**	**MAC GROUP**	**KVVL GROUP**
Mean number of attempts taken for intubation	1.34	1.06

**Table 5:** Comparison of number of attempts taken before successful intubation between the groups

**Number of attempts taken**	**MAC group**	**Percentage (%)**	**KVVL group**	**Percentage (%)**
1^st^ attempt	35	70%	40	80%
2^nd^ attempt	13	26%	10	20%
3^rd^ attempt	2	4%	0	0%
total	50	100%	50	100%
